# Hyaluronate Protects From Benzalkonium Chloride-Induced Ocular Surface Toxicity

**DOI:** 10.1167/tvst.13.10.31

**Published:** 2024-10-21

**Authors:** Alexia Vereertbrugghen, Manuela Pizzano, Florencia Sabbione, Melina S. del Papa, Giselle Rodríguez, María Silvia Passerini, Jeremías G. Galletti

**Affiliations:** 1 Innate Immunity Laboratory, Institute of Experimental Medicine (CONICET/National Academy of Medicine of Buenos Aires), Buenos Aires, Argentina; 2 Medical Affairs, Poen Laboratories, Buenos Aires, Argentina

**Keywords:** corneal nerves, ocular surface epithelium, corneal wound healing, sodium hyaluronate (SH), preservative toxicity

## Abstract

**Purpose:**

The purpose of this study was to investigate the effect of sodium hyaluronate (SH) on benzalkonium chloride (BAK)-induced toxicity in the ocular surface epithelium and corneal nerves.

**Methods:**

Ocular surface epithelial cells from Balb/c mice were cultured with 0.1% to 0.4% SH and 0.001% to 0.01% BAK and their metabolic activity, viability, and wound repair capacity were assessed in vitro. Following a controlled corneal wound, re-epithelialization and recovery of epithelial barrier function and mechanosensitivity were measured in Balb/c mice treated with 0.4% SH 3 times/day and 0.01% BAK twice daily for 3 weeks. Nerve morphology was assessed by confocal microscopy of corneal whole mounts.

**Results:**

Whereas BAK exposure reduced metabolic activity, viability, and wound repair ability of ocular epithelial cells in vitro, pretreatment with SH ameliorated BAK toxicity in a concentration-dependent manner. The highest SH concentration partially reversed the effects of 0.01% BAK in vitro and increased the corneal healing rate of BAK-exposed mice. Although all corneal wounds closed after 4 days, continuous SH treatment improved corneal barrier dysfunction 18 days after wounding and accelerated the recovery of corneal mechanical sensitivity to baseline levels in BAK-exposed mice. SH treatment also increased corneal nerve density in the wounded area after 3 weeks.

**Conclusions:**

SH mitigates BAK-associated ocular epithelial and neurotoxicity in a concentration-dependent manner.

**Translational Relevance:**

Commercially available, high-concentration SH formulations may have added benefits in treating BAK-associated ocular surface toxicity.

## Introduction

Medical glaucoma treatment entails chronic, often lifelong ocular instillation of hypotensive agents in formulations that contain preservatives to reduce the risk of microbial contamination. Benzalkonium chloride (BAK) is the most frequently used preservative in eye drop formulations,^1^ and prolonged exposure to BAK has been linked to ocular surface toxicity.^1^^–^^3^ BAK induces ocular surface disease by promoting corneal and conjunctival epithelial apoptosis,^4^ proinflammatory cytokine secretion,^5^ and conjunctival goblet cell loss,^6^ among other effects.^7^^,^^8^ Despite the development of non-preserved single-use alternatives for some drugs, preserved formulations are still in widespread use due to reduced costs and drug solubility restrictions.^1^^,^^9^^,^^10^

Hyaluronan (or hyaluronic acid) is an extracellular matrix glycosaminoglycan with biological activity. Corneal synthesis of hyaluronan increases during the healing process,^11^ where it favors the adhesion, migration, and proliferation of corneal epithelial cells.^12^^,^^13^ Hyaluronan exerts these effects by interacting with 2 cell surface receptors, CD44 and CD168,^14^^,^^15^ that modulate cellular responses and interactions with other extracellular matrix components. Sodium hyaluronate (SH) is the salt form of hyaluronan, which is commonly used as an ocular surface lubricant in the 0.1% to 0.4% concentration range.^16^ Meta-analyses and a review suggest that SH-based artificial tears may offer some benefits over non-SH-based artificial tears in the treatment of dry eye disease,^16^^–^^18^ and other studies have shown that topical SH treatment improves corneal and conjunctival disease signs associated with BAK toxicity in rabbits^19^^,^^20^ and human patients.^21^ However, whether the biological effects of SH improve other aspects of BAK-induced ocular surface toxicity has not been addressed in such detail.

BAK exposure has well-characterized detrimental effects on both corneal and conjunctival epithelia.^2^^,^^3^^,^^22^^–^^24^ In addition to presenting with an ocular surface disease that resembles inflammatory dry eye syndrome,^25^ patients under long-term treatment with BAK-preserved eye drops have an increased risk of recurrent corneal erosions or persistent epithelial defects due to impaired epithelial healing.^26^ BAK exposure delays corneal wound healing^27^^,^^28^ and it also affects corneal nerves,^29^ either by direct toxicity on the corneal nerve endings^30^ or secondarily due to its well-known negative effects on corneal epithelial cells. On the other hand, SH treatment promotes wound healing in skin keratinocytes by modulating the activity of surface receptors^31^ and it neutralizes BAK-induced toxicity in corneal and conjunctival epithelial cells in vitro.^32^ SH promotes corneal epithelial cell migration in vitro^33^^,^^34^ and the corneal wound healing response in vivo.^34^^–^^36^ However, other studies have suggested that SH and other thickening agents used as lubricants may be detrimental to corneal wound healing in the setting of BAK exposure.^37^^,^^38^ The contradictory results could be related to differences in the dosing schemes that could either favor corneal epithelial cell migration or BAK retention at the ocular surface. Therefore, here, we hypothesized that SH can reduce BAK-associated detrimental effects on the ocular surface when administered prior and sufficiently separate from the BAK-containing formulation. To this aim, we tested different treatment combinations on cultured conjunctival epithelial cells and in a mouse model of corneal wound healing. We found that SH treatment effectively neutralizes in a concentration-dependent manner the toxic effects of BAK on the conjunctival and corneal epithelium and nerves, fostering their recovery after corneal debridement. Thus, our findings support the use of high-concentration SH with appropriate dosing in the management of BAK-induced ocular surface toxicity.

## Materials and Methods

### Cell Cultures and Reagents

All chemical and biological reagents were from Sigma-Aldrich (Buenos Aires, Argentina) unless otherwise specified. All cell cultures were done in RPMI-1640 medium supplemented with 10% fetal calf serum, 10 mM glutamine, 100 U/mL penicillin, 100 µg/mL streptomycin, and 5 × 10^−5^ M 2-mercaptoethanol (complete medium) in a humidified incubator with 5% CO2 at 37°C. A conjunctival epithelial cell line (NAV14) was derived by immortalization of a primary epithelial culture from a Balb/c mouse conjunctival explant.^39^ Epithelial phenotype, monolayer growth, and expression of cytokeratin 13 (conjunctival epithelial marker), and lack of cytokeratin 12 (corneal epithelial marker) were confirmed in the NAV14 clone used in this study. SH was used as a commercially available eye drop formulation (Dropstar LC; Poen Laboratories, Buenos Aires, Argentina) containing 0.4% hyaluronate (Shandong Topscience Biotech, Shandong Province, China) in phosphate-buffered saline (PBS) and no preservatives. The average molecular weight of the SH was calculated as 852 kDa by the supplier from maximum viscosity assessments following the Japanese Pharmacopoeia^40^ (batch SH0420004_1 used in this study). Additionally, other SH batches from the same supplier were measured to be in the 723 to 777 kDa range by size exclusion chromatography.^41^ BAK was obtained as an 80% solution in water from Biopack (Buenos Aires, Argentina).

### In Vitro Model

NAV14 cells were grown until confluent in 96-well plates in complete medium. For the metabolic activity and cell death experiments, the culture medium was replaced with 100 µL of SH in PBS (concentration range = 0.1%–0.4%) or PBS alone as a control, and immediately after, 5 µL of 20-fold concentrated BAK solutions in PBS was added to achieve the final concentrations of 0.001%, 0.005%, and 0.01% BAK. After 15 minutes of incubation, the supernatant was discarded, the cells were washed once with PBS, and then 100 µL of fresh complete medium was added to each well until assaying.

### Metabolic Activity Assay

Resazurin (10 µg/mL, R7017; Sigma-Aldrich) was diluted in complete medium and added immediately after washing the BAK-exposed cells, which were cultured for 2 hours at 37°C. Absorbance at 570 and 600 nm was measured at baseline and at the end of the culture period with a spectrophotometer. Reduction of the oxidized form by live cells was calculated using the formula^42^^,^^43^:

$$\begin{array}{c} \frac{{ɛ}_{OX600nm}\times A_{570nm\;2h}-\varepsilon_{OX570nm}\times A_{600nm\;2h}}{{ɛ}_{RED570nm}\times A_{600nm\;t0}-\varepsilon_{RED600nm}\times A_{570nm\;t0}} \end{array}$$

where ε_*OX*_ and ε_*RED*_ correspond to the molar extinction coefficients of the oxidized (80,586 and 117,216 for 570 and 600 nm, respectively) and reduced (155,677 and 14,652 for 570 and 600 nm, respectively) forms of resazurin and *A* corresponds to the absorbance at the indicated wavelengths (570 and 600 nm) and time points, respectively. Reduction percentages are expressed relative to control cells.

### Cell Death Assay

Release of lactate dehydrogenase (LDH) from dead cells was quantified using a CyQUANT LDH Citotoxicity Assay Kit (C20300; Invitrogen, Buenos Aires, Argentina) per the manufacturer's protocol. In brief, after the 2-hour incubation period, 50 µL of the cell supernatant was collected and mixed with 50 µL of the reaction mixture, incubated at room temperature for 30 minutes protected from light, then 50 µL of the stop solution was added, and finally absorbance at 490 and 680 nm was measured. To determine LDH activity, background (680 nm) absorbance was subtracted from the 490-nm absorbance.

### Scratch Wound Healing Assay

Cell monolayers were exposed to SH and BAK as described above. At the end of the 15-minute exposure and before washing the cells, a controlled wound was generated by gently scratching the monolayer with a 200-µL pipette tip.^44^^,^^45^ The cells were then washed and replenished with fresh culture medium. Using an inverted microscope, micrographs were taken at 0, 4, 8, and 24 hours after scratching and the resulting images were later analyzed by a blind observer using ImageJ software (version 2.15.0, https://imagej.net/software/fiji/). At least five estimates of wound width were taken per micrograph and averaged as one data point, and the experiment was performed twice with six replicates per condition. For some experiments, cells were treated with mitomycin C 10 µg/mL for 2 hours before scratching.

### Mice

Balb/c (BALB/cAnNCrl) mice were originally obtained from Charles River Laboratories (Wilmington, MA, USA). Mice were bred and maintained at the Institute of Experimental Medicine’s animal facility. All mice were 8 weeks old at the beginning of the experiments and both male and female mice were included. All protocols were approved by the Institute of Experimental Medicine animal ethics committee and adhered to the Association for Research in Vision and Ophthalmology Statement for the Use of Animals in Ophthalmic and Vision Research. A total of 88 mice were used in this study.

### In Vivo Model

Mice were given 0.4% SH or PBS eye drops (5 µL/eye) 3 times a day (8 AM, 2 PM, and 8 PM) and 0.01% BAK or PBS eye drops (5 µL/eye) twice a day (10 minutes after the first and last daily SH administrations). Two days after starting the eye drop administrations, mice were anesthetized with an intraperitoneal (IP) injection of ketamine (100 mg/kg) and xylazine (10 mg/kg) and placed on a heated pad. After a 0.5% proparacaine eye drop was applied on the right eye, a 2-mm corneal wound was created by removing the epithelium using an Alger brush with a 1-mm rotating burr. After washing the ocular surface with PBS to remove epithelial debris, the resulting corneal wound was stained with fluorescein and photographed to record its baseline dimensions as described below. Finally, the eyes were kept moist with PBS drops until the mouse fully recovered from anesthesia.

### Assessment of Corneal Wound Size

Corneal wound size was determined by modifying a previously described technique.^46^^,^^47^ In brief, 0.5 µL of 0.25% fluorescein (Fluoresceína; Poen Laboratories) was applied to the right eye and then a 10-second-long video of the right eye under blue light was captured with the aid of a fluorescence-adapted dissection microscope (NightSea SFA-RB; Electron Microscopy Sciences, Hatfield, PA, USA). For analysis, a masked observer exported a representative video frame as an image to ImageJ software (version 2.15.0, https://imagej.net/software/fiji/). First, the total corneal area was calculated by adjusting a circle to the horizontal intercanthal diameter (which is not affected by the extent of the palpebral opening), and then the fluorescein-stained wounded area was measured by fitting the largest polygonal shape possible to its contour. Finally, the wounded area was expressed as a fraction of the total corneal area. Wound area measurements were taken every 12 hours for up to 96 hours.

### Assessment of Corneal Epithelial Barrier Function

Corneal fluorescein uptake was measured as previously described.^46^^,^^47^ In brief, 0.5 µL of dextran-fluorescein isothiocyanate (average molecular weight 3000–5000, 10 mg/mL in PBS) was applied to the right eye and then the mouse was returned to its cage. After 3 minutes, a 10-second-long video of the right eye under blue light was captured with the aid of a fluorescence-adapted dissection microscope described above. For analysis, a masked observer exported a representative video frame as an image and selected the corneal area suitable for analysis, excluding reflections and other artifacts, using ImageJ software. Then, the green channel was extracted and the mean fluorescence intensity within the resulting region of interest was calculated after background subtraction (50-pixel rolling ball radius), and the average of both eyes was used for analysis.

### Assessment of Corneal Mechanical Sensitivity

Mechanical thresholds were determined using a mouse-adapted version of Cochet-Bonnet esthesiometry.^46^^–^^48^ Nylon 6-0 monofilament was cut into segments of varying lengths (1.0 to 5.5 cm in 0.5 cm steps). With the mouse held firmly in one hand, the right cornea was touched six times with each filament, starting with the longest segment. A positive response was defined as blinking and retraction of the eye in reaction to at least three of the six tries. The longest segment yielding a positive response was used as the sensitivity threshold.

### Collection of Eye Tissue

After euthanasia, enucleation was performed by gently proptosing the right eye globe and cutting the optic nerve with curved scissors. Eyes were collected in ice-cold fixative solution as described by Tadvalkar et al.^49^ In brief, eyes were fixed in a pre-chilled formaldehyde-containing buffer for 75 minutes, washed, and stored in methanol at −20°C until processed for staining. Then, the fixed corneas were cut from the back of the eye under a dissection microscope, permeabilized with a graded methanol-Triton X-100 series, blocked overnight with 1% bovine serum albumin and 1% goat serum in PBS, and stained overnight with Alexa 488-conjugated anti-tubulin β3 antibody (801203; BioLegend). Each batch of anti-tubulin β3 antibody was titrated before use to minimize background staining, usually resulting in 0.5 to 0.7 µL antibody/200 µL buffer/cornea (2.5–3.5 µg/mL) as optimal. The stained corneas were washed 3 times for 60 minutes in PBS-Tween 0.02%, counterstained with 1 ug/mL DAPI, mounted flat with the aid of relaxing cuts in Aqua-Poly/Mount (PolySciences), and stored at 4°C until imaged.

### Confocal Laser Scanning Microscopy Acquisition

Image acquisition was performed with a FluoView FV1000 confocal microscope (Olympus, Tokyo, Japan) equipped with Plapon 60X/1.42 and UPlanSapo 20X/0.75 objectives. Z stacks (1-µm step size) spanning the entire corneal epithelium and the anterior stroma (approximately 45 µm) were obtained first at the corneal center (defined as the center of the nerve whorl or as the center of the disorganized area in those samples with highly disrupted nerve whorls), and then at 2 opposite locations 600 µm from the center. Corneal nerve analysis was performed with the Sholl plugin in ImageJ software. In brief, a maximum intensity projection of all the sections encompassing the corneal sub-basal nerve mat and the anterior stromal nerves was created, then the background was subtracted (50-pixel rolling ball radius), and the image was thresholded. Finally, 10 concentric circles with a 10-µm radius step size were traced at the center of the final image, and the resulting sum of intersections of tubulin β3^+^ nerves for each concentric circle was calculated using the software and used for analysis.^50^

### Statistical Analysis

Student's *t*-test and 1-way or 2-way analysis of variance (ANOVA) with Dunnet's or Sidak's post hoc tests were used to compare the means of 2 or more samples, respectively. Significance was set at *P* < 0.05 and 2-tailed tests were used in all experiments. Calculations were performed using GraphPad Prism version 9 software (GraphPad Software, La Jolla, CA, USA).

## Results

### SH Protects Ocular Surface Epithelial Cells From BAK Toxicity In Vitro

In order to test the effect of SH on ocular BAK toxicity, we first modeled in vitro the exposure of the ocular surface to this preservative. To this aim, we resorted to the NAV14 cell line, an immortalized murine conjunctival epithelial cell line derived from the Balb/c mouse strain. Confluent monolayer cultures were exposed first to different sodium hyaluronate concentrations (0.1%, 0.2%, 0.3%, and 0.4%), and immediately after, BAK was added to the culture medium to achieve different final concentrations (0.001%, 0.005%, and 0.01%). The highest point in the BAK curve was defined based on the most commonly found preservative concentration in commercially available formulations (0.01%), and to account for reflex tearing and tear clearance, 2-fold (0.005%) and 10-fold (0.001%) dilutions were also tested. The 15-minute exposure time was based on the average tear turnover rate (15%/min) reported by fluorophotometry^51^ to ensure that the highest preservative concentration condition surpassed the exposure of a patient's eye receiving one drop of 0.01% BAK-containing medication. After the exposure was completed, the BAK dilution was replaced with fresh medium for further testing after 2 hours in culture.

**Figure 1.**
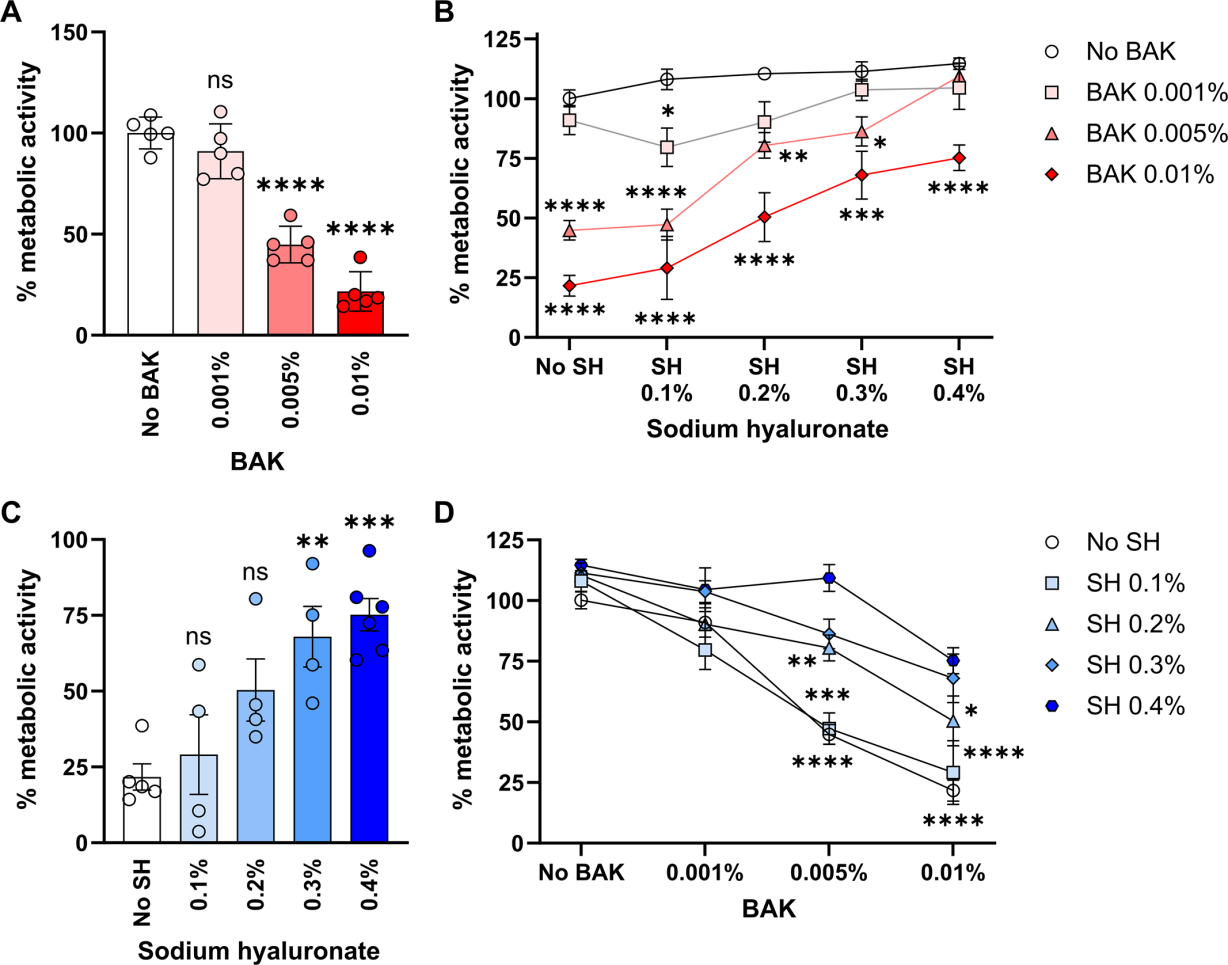
**Effect of sodium hyaluronate on the metabolic activity of ocular surface epithelial cells exposed to benzalkonium chloride.** Confluent NAV14 cell monolayers were exposed to different concentrations (0.1–0.4%) of sodium hyaluronate (SH) and then exposed to different concentrations (0.001–0.01%) of benzalkonium chloride (BAK) for 15 minutes. Then, the BAK-containing medium was removed and the cells were cultured for 2 hours in the presence of fresh medium with resazurin to quantify metabolic activity (expressed relative to control cells). (**A**) Effect of different BAK concentrations on metabolic activity in the absence of SH. (**B**) Effect of different BAK concentrations on metabolic activity in the presence of increasing SH concentrations. (**C**) Effect of increasing SH concentrations on metabolic activity of cells exposed to BAK 0.01%. (**D**) Effect of increasing SH concentrations on metabolic activity of cells exposed to increasing BAK concentrations. For all panels, the mean ± SEM from at least 3 independent experiments is shown, and 1-way or 2-way ANOVA was used to compare means with Dunnet's post hoc test. * Indicates *P* < 0.05, ** indicates *P* < 0.01, *** indicates *P* < 0.001, **** indicates *P* < 0.0001, and ns indicates not significant.

**Figure 2.**
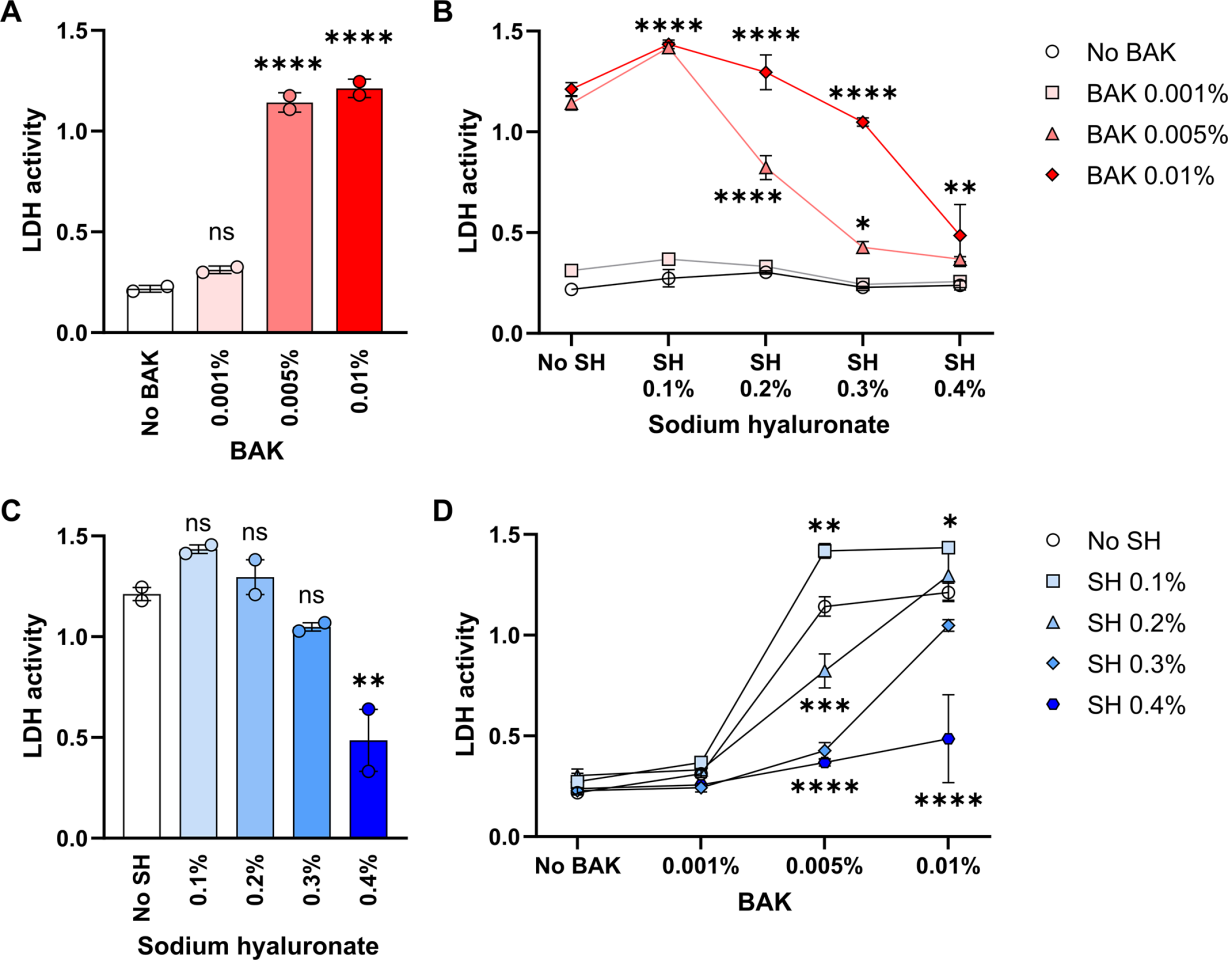
**Effect of sodium hyaluronate on the death of ocular surface epithelial cells exposed to benzalkonium chloride.** Confluent NAV14 cell monolayers were exposed to different concentrations (0.1–0.4%) of sodium hyaluronate (SH) and then exposed to different concentrations (0.001–0.01%) of benzalkonium chloride (BAK) for 15 minutes. After washing, cells were cultured for 2 hours until the release of lactate dehydrogenase (LDH) to the supernatant was quantified. (**A**) Effect of different BAK concentrations on LDH release in the absence of SH. (**B**) Effect of different BAK concentrations on LDH release in the presence of increasing SH concentrations. (**C**) Effect of increasing SH concentrations on LDH release from cells exposed to BAK 0.01%. (**D**) Effect of increasing SH concentrations on LDH release from cells exposed to increasing BAK concentrations. For all panels, mean ± SEM from a representative experiment (*n* = 2) is shown, and 1-way or 2-way ANOVA was used to compare means with Dunnet's post hoc test. * Indicates *P* < 0.05, ** indicates *P* < 0.01, *** indicates *P* < 0.001, **** indicates *P* < 0.0001, and ns indicates not significant.

In the first assay, reduction of resazurin (added in the fresh culture medium) was measured as an indicator of metabolic activity. BAK showed a concentration-dependent toxic effect (reduction in metabolic activity) on ocular epithelial cells in vitro (Fig. 1A). As shown in Figure 1B, BAK toxicity in this system was reduced in the presence of SH. We observed a concentration-dependent protective effect of SH in the 0.1% to 0.4% range, which was maximal in the presence of 0.01% BAK. As depicted in Figure 1C, adding 0.4% SH led to a 3-fold increase in the metabolic activity of ocular epithelial cells exposed to 0.01% BAK. Accordingly, 0.1% SH offered little protection from BAK toxicity in this assay while 0.4% SH afforded the greatest improvement in metabolic activity (Fig. 1D). In the second assay, the release of intracellular lactate dehydrogenase to the culture supernatant was measured as an indicator of cell death. In line with the first assay, BAK-induced cell death was concentration-dependent (Fig. 2A). Although BAK 0.001% induced little or no LDH release above baseline, exposure to either BAK 0.005% or BAK 0.01% led to a significant increase in cell death. In addition, pretreatment with SH afforded concentration-dependent protection from BAK-induced cell death (Fig. 2B). Of note, only SH 0.4% reduced cell death induced by the highest BAK concentration (Fig. 2C) whereas SH 0.1% was not protective at all (Fig. 2D). Altogether these results show that SH pretreatment reduces BAK-associated toxicity in ocular epithelial cells in vitro.

### SH Promotes the In Vitro Wound Healing Response of Ocular Surface Epithelial Cells Exposed to BAK

**Figure 3.**
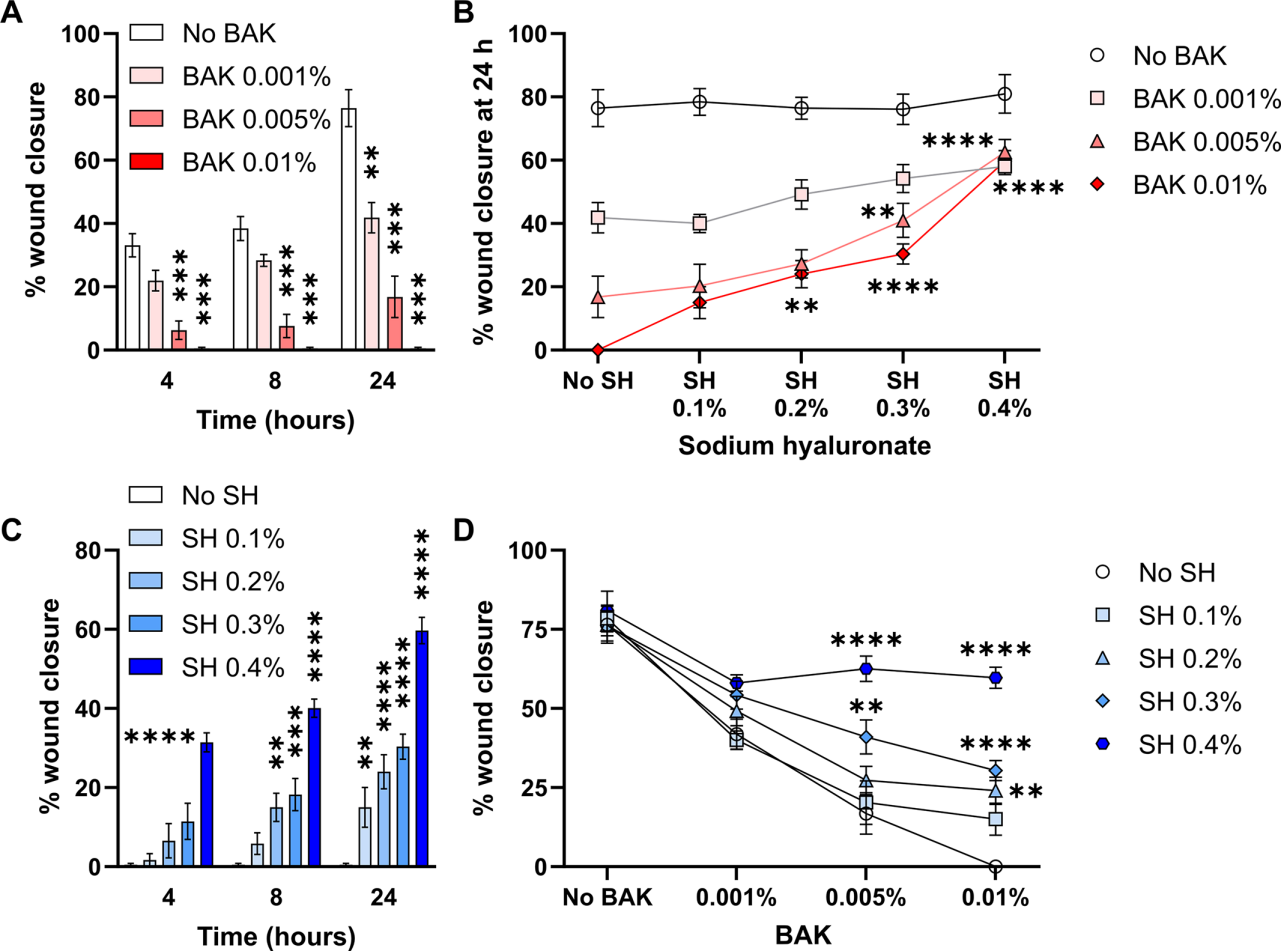
**Effect of sodium hyaluronate on the healing response of ocular surface epithelial cells exposed to benzalkonium chloride.** Confluent NAV14 cell monolayers were exposed to different concentrations (0.1–0.4%) of sodium hyaluronate (SH) and then exposed to different concentrations (0.001–0.01%) of benzalkonium chloride (BAK) for 15 minutes. After induction of a controlled scratch wound, the cells were monitored over 24 hours for quantification of the healing response. (**A**) Effect of different BAK concentrations on the wound healing response in the absence of SH. (**B**) Effect of different BAK concentrations on the healing response in the presence of increasing SH concentrations. (**C**) Effect of increasing SH concentrations on the healing response of cells exposed to BAK 0.01%. (**D**) Effect of increasing SH concentrations on the wound healing response of cells exposed to increasing BAK concentrations. For all panels, mean ± SEM from 2 independent experiments with 4 to 6 replicates per condition is shown, and 1-way or 2-way ANOVA was used to compare means with Dunnet's post hoc test. ** Indicates *P* < 0.01, *** indicates *P* < 0.001, and **** indicates *P* < 0.0001.

After observing in vitro protection from BAK toxicity, we tested whether SH pretreatment had an effect on a more physiological cell response. To this aim, we performed a scratch wound healing assay, which measures both the migration and proliferation of cells.^52^ Cell monolayers were pretreated with SH and exposed to BAK as detailed in Figures 1 and 2, and then a controlled scratch wound was induced in each culture well. The extent of wound closure was measured after 4, 8, and 24 hours (Supplementary Fig. 1). As shown in Figures 3A and 3B, BAK exposure delayed the wound closure rate at every time point in a concentration-dependent fashion. Of note, BAK 0.01% completely abrogated the healing response in cultured ocular surface epithelial cells, in line with the marked reduction of metabolic activity and extensive cell death observed in the previous experiments (see Fig. 3B). In addition, in agreement with previous findings, pretreatment with SH showed a concentration-dependent protection from the negative effect of BAK exposure on wound healing (see Figs. 3B, 3C, 3D). Whereas the lower SH concentrations had little effect on the toxic effect of the highest BAK exposure, SH 0.4% pretreatment restored the healing response of BAK 0.01%-exposed cells from nil to 60% at 24 hours (as reference, the healing rate of non-BAK-exposed cells at the same time point was 81%). Of note, mitomycin C-pretreatment of cells to prevent their proliferation did not significantly modify the effect of SH and/or BAK exposure on wound healing (Supplementary Fig. S2). Altogether, these results demonstrate that SH pretreatment favors the healing response of BAK-exposed cells in vitro.

### SH Fosters Corneal Epithelial Repair In Vivo in the Presence of BAK

**Figure 4.**
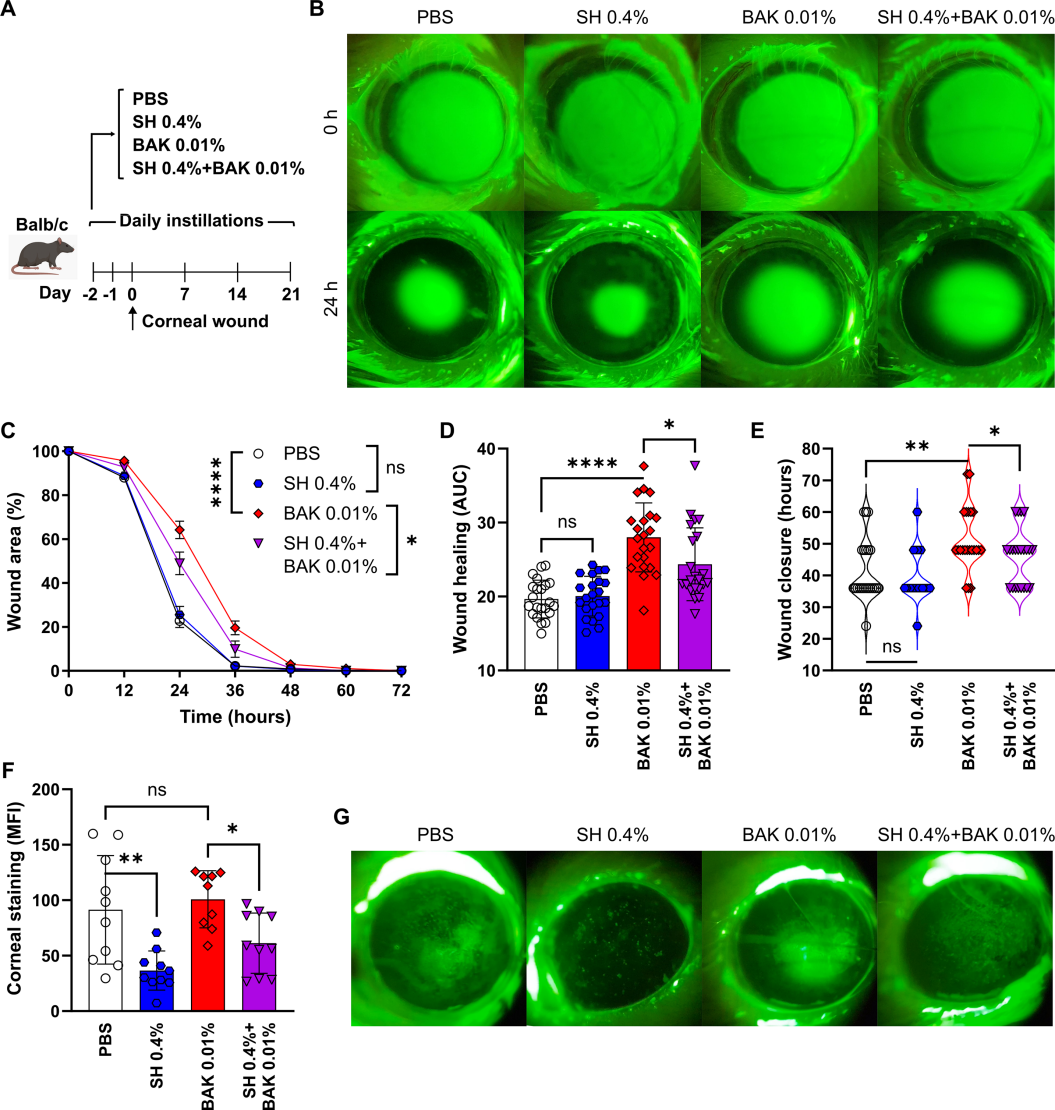
**Effect of sodium hyaluronate on corneal wound healing in mice exposed to benzalkonium chloride.** (**A**) Experimental design: the healing of a corneal wound over 21 days was monitored in mice treated with either saline (PBS) or 0.4% sodium hyaluronate (SH) 3 times/day and either PBS or 0.01% benzalkonium chloride (BAK) twice daily (4 groups, *n* = 22/group). (**B**) Representative micrographs from each group of the fluorescein-stained corneas at baseline (0 hours) and 24 hours post-wounding. (**C**) Quantification of wound size (relative to starting wound area for each mouse) over time. (**D**) Area under the wound size/time curve (estimate of healing rate) from one representative experiment. (**E**) Time (hours) until full wound closure for each mouse from one representative experiment. (**F**) Quantification (mean fluorescence intensity [MFI], one representative experiment) and (**G**) representative micrographs of corneal uptake of fluorescent dextran on day 18 post-wounding. For all panels, mean ± SEM is shown, and 1-way or 2-way ANOVA was used to compare means with Dunnet's post hoc test. * Indicates *P* < 0.05, ** indicates *P* < 0.01, **** indicates *P* < 0.0001, and ns indicates not significant.

Because we observed that SH 0.4% afforded the greatest protection from the toxic effects of BAK exposure on ocular surface epithelial cells in vitro, we validated these findings in a preclinical in vivo model (Fig. 4A). Mice were pretreated topically in one eye for 2 days with either saline or SH 0.4% 3 times a day combined with either saline or BAK 0.01% twice daily (4 treatment groups), and then a controlled corneal wound was created in one eye. The topical treatment continued for 3 weeks to monitor the corneal healing response (Fig. 4B). As shown in Figure 4C, mice treated with either saline or SH 0.4% showed the fastest corneal epithelial healing response, with no statistically significant difference between the 2 groups as assessed by the healing rate (Fig. 4D) or the time to complete wound closure (Fig. 4E). By contrast, BAK-exposed mice exhibited the slowest healing response as assessed by any metric while SH + BAK-treated mice had a significantly improved corneal epithelial healing response compared to BAK-treated mice (see Figs. 4C, 4D, 4E). Of note, the protection afforded by SH over BAK toxicity was incomplete, as SH + BAK-treated mice had slower healing responses than non-BAK-exposed mice by all metrics. All corneal wounds were completely healed after 72 hours in all treatment groups. Nonetheless, we measured the corneal uptake of fluorescein-tagged dextran as an indicator of corneal epithelial integrity 18 days after wounding because mice experience recurrent corneal erosions in this model.^53^^,^^54^ Both saline- and BAK-treated mice showed comparably high dye uptake (Figs. 4F, 4G). Of note, the addition of SH to the treatment of saline- and BAK-exposed mice significantly improved corneal epithelial barrier function as evidenced by the decrease in dye uptake (see Figs. 4F, 4G). Altogether these findings show that SH treatment promotes the corneal epithelial healing response to injury in the BAK-exposed eyes of mice.

### SH Favors Corneal Nerve Recovery After Wounding in the Presence of BAK

**Figure 5.**
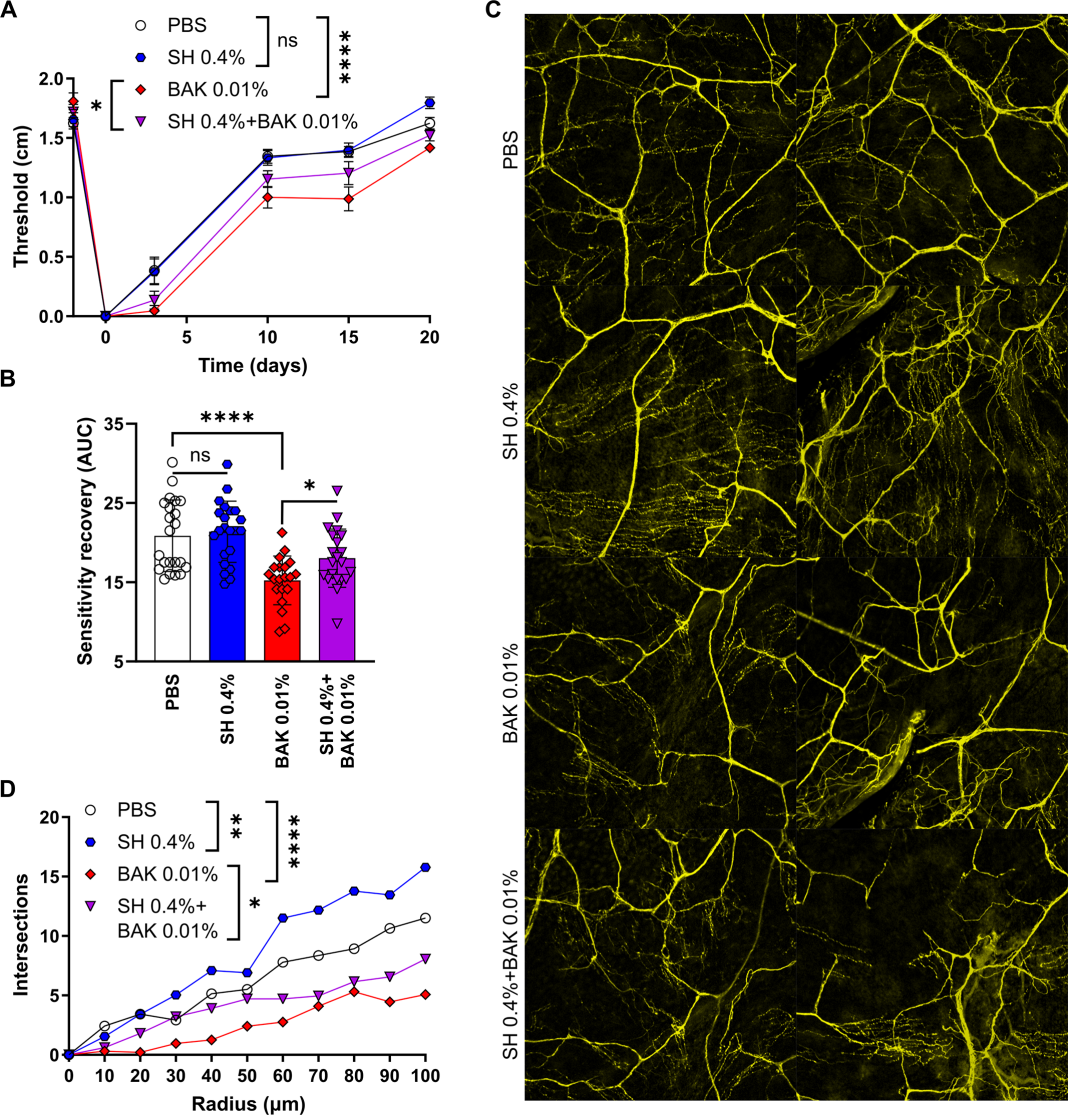
**Effect of sodium hyaluronate on corneal nerve regeneration in mice exposed to benzalkonium chloride.** (**A**) Corneal mechanosensitivity thresholds were determined on the specified days of the corneal wound healing experiment described in Figure 4A. (**B**) Area under the mechanosensitivity threshold/time curve (estimate of mechanosensitivity recovery rate) from one representative experiment. (**C**) Representative corneal whole-mount micrographs spanning the sub-basal and anterior stromal nerves from all treatment groups. (**D**) Quantification of nerve density in corneal whole-mounts from all treatment groups as determined by Sholl analysis over 10 concentric circles of increasing radii in 10 µm steps. For all panels, mean ± SEM is shown, and 1-way or 2-way ANOVA was used to compare means with Dunnet's post hoc test. * Indicates *P* < 0.05, ** indicates *P* < 0.01, **** indicates *P* < 0.0001, and ns indicates not significant.

As corneal nerves are pillars of ocular surface homeostasis, we examined the effect of SH treatment on corneal nerve regeneration after wounding. First, we recorded the corneal mechanical sensitivity threshold as an indicator of corneal nerve function using a modified Cochet-Bonnet esthesiometry technique.^46^^–^^48^ Corneal mechanosensitivity dropped to nil immediately after wounding, which is consistent with the complete ablation of corneal intraepithelial nerves during epithelial debridement (Fig. 5A). Both saline- and SH-treated mice recovered the baseline mechanosensitivity threshold after 3 weeks, with no statistically significant difference in the recovery rate between the 2 despite a positive trend for SH-treated mice (Fig. 5B). In line with previous findings, BAK-treated mice regained corneal mechanosensitivity at a slower rate and did not recuperate in full after 3 weeks (compared to their baseline level). By contrast, SH + BAK-treated mice recovered at a significantly faster pace (see Figs. 5A, 5B). Three weeks after wounding, we analyzed the morphology of corneal nerves. All groups showed normal stromal nerves from which intraepithelial corneal nerves sprouted in a disorganized pattern consistent with regeneration (Fig. 5C). However, focal areas without intraepithelial nerve sprouting were more evident in BAK-treated mice. Quantification of nerve complexity by Sholl analysis (Fig. 5D) revealed that corneal nerve density was greater in SH-treated mice than in saline-treated mice while it was significantly impaired in BAK-treated mice. In agreement with the functional testing data, nerve density was significantly higher in SH + BAK-treated mice than in BAK-treated mice, although it did not reach the levels of saline-treated mice. Altogether these findings show that SH treatment promotes corneal nerve regeneration after injury in the BAK-exposed eyes of mice.

## Discussion

Preservative-induced ocular surface toxicity is a significant clinical challenge, as many patients experience the desired reduction in intraocular pressure but at the cost of ocular surface symptoms that impact their quality of life and jeopardize their treatment adherence.^25^ Topical administration of SH is routinely used in dry eye therapy and it has also been proposed as a useful agent in the management of ocular toxicity induced by BAK, the most commonly used preservative in eye drops.^19^^,^^21^ However, other studies have suggested the opposite,^37^^,^^38^ which may be related to differences in SH composition, concentration, and administration of the formulations tested.^41^ Here, we show that high-concentration SH is effective at neutralizing BAK-induced toxicity when administered before the preservative as it improves conjunctival epithelial cell viability in vitro and fosters corneal epithelial and nerve regeneration in vivo.

There is considerable heterogeneity in SH formulations approved for ocular use,^41^ which stems from the fact that hyaluronan is a single-chain polymer containing any number of the same disaccharide units.^55^^,^^56^ Because the native hyaluronan in healthy solid tissues and synovial fluid is mostly high molecular weight (>1000 kDa),^57^^,^^58^ the low molecular weight designation in the literature comprises anything from 10 disaccharide units up to 1000 kDa.^55^ Fragments smaller than 10 disaccharide units are termed SH oligosaccharides and exhibit less affinity for CD44.^59^ Fragment size has a considerable bearing on the effects of SH, which may be quite the opposite.^34^^,^^60^ High molecular weight SH has anti-inflammatory effects and favors tissue repair.^58^^,^^61^ On the other hand, SH oligosaccharides, which result from hyaluronan catabolism by hyaluronidases and oxidative stress, serve as danger signals to the immune system as they activate Toll-like receptors 2 and 4 and promote inflammation.^60^^,^^62^^,^^63^ The SH formulation tested here had an average molecular weight of 852 kDa, which is lower than that of hyaluronan in healthy synovial fluid and cartilage (>1000 kDa) but higher than in serum (100–300 kDa).^57^^,^^64^ This molecular size lies within the moderate range described in some studies (500–800 kDa)^57^^,^^58^ and roughly coincides with that measured in the aged human and bovine vitreous.^65^ However, our findings are in line with the anti-inflammatory and pro-regenerative effects associated with high molecular weight SH,^33^^–^^36^^,^^66^^,^^67^ probably because a molecular size smaller than 500 kDa seems to be required for the proinflammatory effects of low molecular weight SH to be observed.^58^ Consistently, a recent report by Lin et al. specifically described how fragmentation of high molecular weight hyaluronan (2670 kDa) is accompanied by the loss of its positive effects on corneal epithelial wound healing.^34^

We also observed a concentration-dependent effect of 0.1% to 0.4% SH on ocular epithelial cell metabolic activity and migration in vitro. A prospective interventional study on patients undergoing pterygium surgery reported faster corneal epithelial healing in those receiving 0.3% SH (molecular weight = 1000 kDa) than in those treated with 0.1% SH (molecular weight 1200 kDa).^67^ Seino et al. found that 0.1% and 0.3% SH (molecular weight = 1000 kDa) had a similar concentration-dependent effect in corneal epithelial cell cultures and organ-cultured corneal epithelium.^37^ Our in vitro findings in the presence of BAK also agree with theirs, but our in vivo findings starkly contrast with their in vivo wound healing data in the presence of BAK. The opposite outcomes probably result from the considerable differences between models. Whereas the one explored by Seino et al.^37^ involved 4 topical administrations of 0.02% BAK or 0.3% SH immediately every 2 hours starting right after corneal wounding, ours involved continuous administration of SH 0.4% 3 times per day and BAK 0.01% 2 times per day starting 2 days before corneal wounding and ending 3 weeks later. In our study, SH was delivered to the ocular surface at least 10 minutes before BAK to simulate the interval recommended to patients receiving two different eye drop formulations.^68^ By contrast, the results from Seino et al.^37^ coincide with those from another study in which ocular administration of a solution containing BAK and either methylcellulose, carboxymethylcellulose, or hydroxypropyl methylcellulose delayed corneal wound healing compared to BAK alone.^38^ Thus, SH may increase the ocular surface retention of BAK when administered simultaneously or sequentially, and therefore it may be detrimental to corneal wound healing under such a dosing scheme. By contrast, our results suggest that when administered at least 10 minutes after BAK, SH does indeed enhance corneal epithelial regeneration.

In addition to the short-term effects of continuous SH treatment upon corneal wounding, our study suggests that extending the administration beyond the closure of the corneal epithelial defect benefits ocular surface homeostasis (see Figs. 4, 5). The full restoration of the corneal epithelial cell layer implies the reformation of the hemidesmosomes that anchor the cells to the basement membrane, a process that involves immune cell recruitment and takes several weeks in mice.^53^^,^^69^ The reduced incidence of recurrent corneal erosions and the improved corneal epithelial barrier function observed in mice treated with SH irrespective of BAK exposure is in line with previous reports of improved corneal epithelial wound healing in vitro and in vivo.^19^^–^^24^^,^^33^^–^^35^^,^^37^^,^^70^^–^^73^ SH affords protection against the increased oxidative stress induced by BAK and other preservatives, such as thimerosal.^32^^,^^74^ Our study extends these findings to corneal reinnervation after injury, with improved corneal nerve function induced by SH in BAK-exposed mice (see Figs. 5A, 5B) and higher nerve density in both BAK- and non-BAK-exposed mice (see Fig. 5D). The mechanism by which SH treatment improves corneal reinnervation remains undetermined. Considering its beneficial action on corneal epithelial repair and the intimate relationship between corneal epithelial cells and nerve fibers,^75^^,^^76^ the positive neural effects of SH treatment could be secondary to faster or better restoration of the corneal epithelial extracellular matrix. Alternatively, because corneal nerves are highly sensitive to inflammatory changes,^46^^–^^48^^,^^50^^,^^77^ it is also possible that the positive neural effects of SH treatment are due to its anti-inflammatory actions.^19^ In line with the latter option, increased transient receptor potential vanilloid-1 channel activity in corneal nerves in the context of ocular surface inflammation propagates axonal degeneration^47^ whereas SH modulates the opening of these channels in sensory neurons.^78^ Of note, hyaluronate also promotes neuronal regeneration or survival in the retina^79^^,^^80^ and dorsal root ganglion cultures.^81^

This study has limitations. First, although our data show that SH administration has positive effects on conjunctival and corneal epithelial and neural regeneration, the underlying mechanism remains unknown. As the positive influence on corneal epithelial and neural recovery was more marked in BAK-exposed mice, this might indicate that more than one mechanism is at work, for example, CD44-dependent, BAK-independent signaling, and neutralization of BAK-induced P2 × 7 activation. More work is warranted in this direction. Second, our in vitro model uses a conjunctival epithelial cell line and thus some of the in vitro findings may not apply to the corneal epithelium. Several studies have shown that hyaluronate ameliorates BAK toxicity on corneal epithelial cells in vitro but the evidence on conjunctival epithelial toxicity is more limited.^12^^,^^13^^,^^22^^,^^23^^,^^37^ Our report complements these studies by showing that SH also mitigates BAK toxicity on conjunctival epithelial cells in vitro. Conjunctival toxicity per se is a significant aspect of BAK-induced ocular surface disease.^1^^–^^3^ Third, our findings are derived from in vitro and in vivo preclinical models and do not necessarily translate to patients. Nonetheless, SH has shown a positive corneal neuro-regenerative effect in patients with severe dry eye, albeit in a small sample study.^82^ Our data are in line with this report and suggests that this action might extend to BAK-induced ocular surface toxicity on corneal epithelial cells and nerves. Studies using other preclinical models of ocular surface disease that present with corneal nerve damage, such as dry eye,^76^ or involving patients treated with SH formulations and followed using noninvasive in vivo corneal confocal microscopy might yield additional information. At any rate, our work supports the widely approved indication of SH treatment for ocular surface disease and suggests that it might be particularly beneficial in the context of BAK exposure.

## Supplementary Material

Supplement 1

Supplement 2
