# Supplementary material for: Hyaluronate Protects From Benzalkonium Chloride-Induced Ocular Surface Toxicity
Source: Transl Vis Sci Technol. 2024 Oct 21;13(10):31. doi: 10.1167/tvst.13.10.31 (PMC11498636; doi:10.1167/tvst.13.10.31)
Supplement: Supplement 1 [file tvst-13-10-31_s001.pdf]

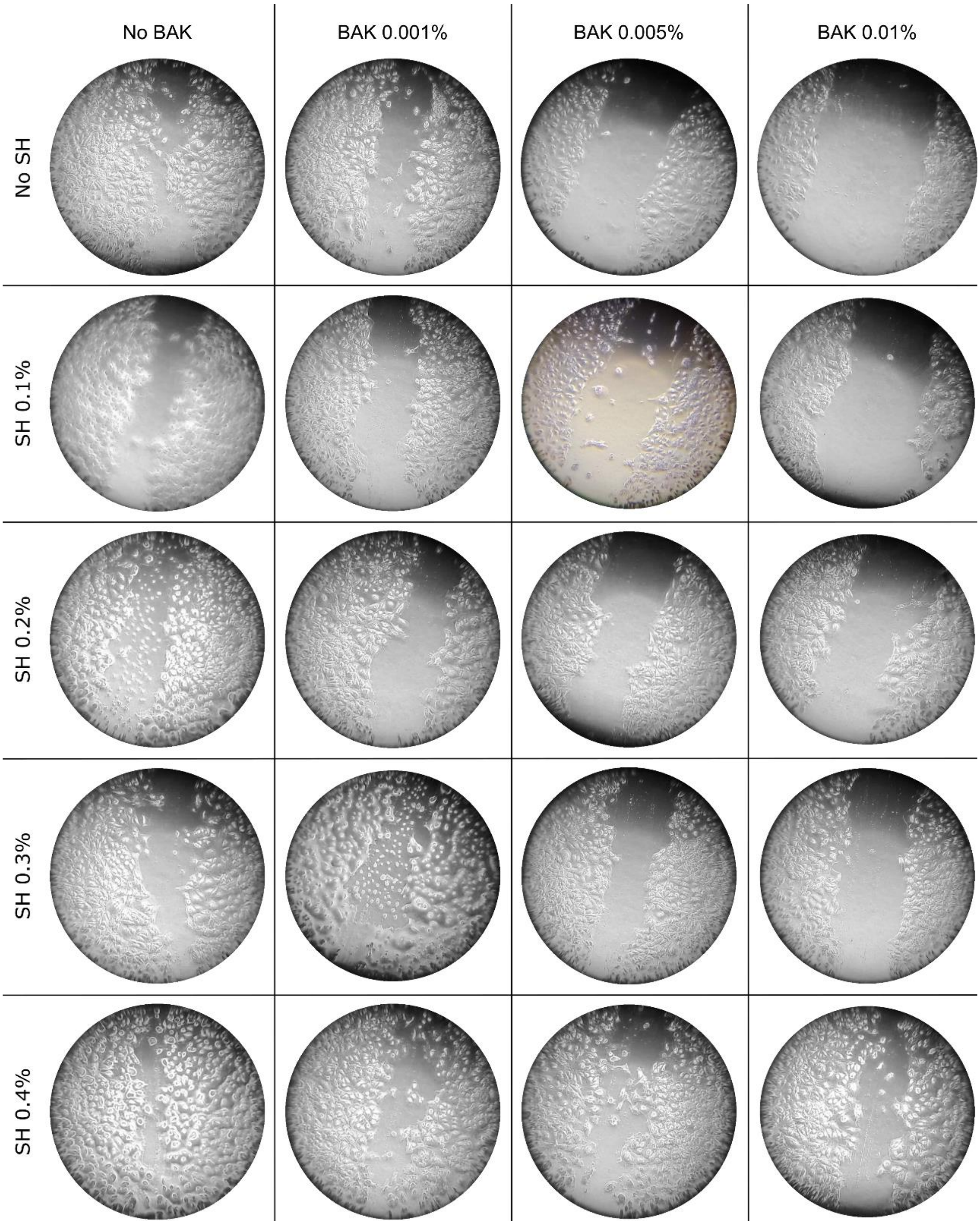

Supplementary Figure 1 - Effect of sodium hyaluronate on the healing response of ocular surface epithelial cells exposed to benzalkonium chloride. Confluent NAV14 cell monolayers were exposed to different concentrations (0.1-0.4%) of sodium hyaluronate (SH) and then exposed to different concentrations (0.001-0.01%) of benzalkonium chloride (BAK) for 15 min. After induction of a controlled scratch wound, cells were monitored over 24 h for quantification of the healing response. Representative micrographs of cultured cells at 24 h post-wounding are shown.
