# Supplementary material for: Hyaluronate Protects From Benzalkonium Chloride-Induced Ocular Surface Toxicity
Source: Transl Vis Sci Technol. 2024 Oct 21;13(10):31. doi: 10.1167/tvst.13.10.31 (PMC11498636; doi:10.1167/tvst.13.10.31)
Supplement: Supplement 2 [file tvst-13-10-31_s002.pdf]

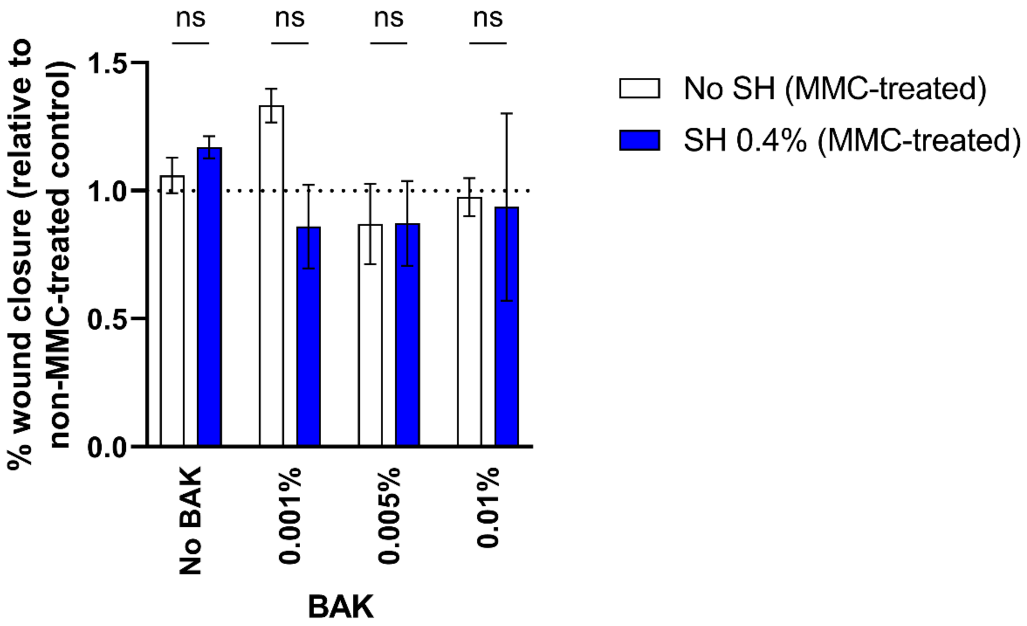

Supplementary Figure 2 - Effect of mitomycin C pre-treatment on the healing response of ocular surface epithelial cells exposed to sodium hyaluronate and benzalkonium chloride. Confluent NAV14 cell monolayers were pre-treated with 10 µg/ml mitomycin C (MMC), then exposed to different concentrations (0.1-0.4%) of sodium hyaluronate (SH) and finally exposed to different concentrations (0.001-0.01%) of benzalkonium chloride (BAK) for 15 min. After induction of a controlled scratch wound, cells were monitored over 24 h for quantification of the healing response. Wound closure rates for each MMC-treated condition are shown relative to the corresponding non-MMC-treated condition (same SH and BAK concentrations) as mean±SEM from two independent experiments with 4-6 replicates per condition is shown. Two-way ANOVA was used to compare means with Dunnet’s post-hoc test. ns indicates not significant.
